# Relative Merits of Watt's Crystal Gold among Filling Materials

**Published:** 1884-12

**Authors:** J. F. P. Hodson

**Affiliations:** New York.


ARTICLE III.

### A CONSIDERATION OF THE RELATIVE MERITS OF WATTS' CRYSTAL GOLD AMONG FILLING MATERIALS.

BY J. F. P. HODSON, D. D. S., NEW YORK.

**(Read before the New England Dental Society, at its Twenty-Second Annual meeting, held in Boston, Oct. 2 and 3, 1884.)**

*Mr. President and Gentlemen*—It is with much diffidence, and yet with a lively sense of the honor you have conferred upon me in extending to me the special and very kindly invitation to appear before you, that I am here today, and, while thanking you for the distinguished courtesy of which I am the object, cannot but feel how very greatly you might have bettered the selection of your object than your subject. For, very truly, there are few, if indeed there be any, which ought more properly to engage our most serious consideration, than the comparative merits of filling materials for the teeth, as few I think will question, that whatever our surgical or other dental skill or ability, the perfect stopping is, in a very real sense, the *ultima thule* of all dentistry.

As, however, it has been very delicately hinted to me by your committee that your especial hunger is for practical facts, rather than theoretical disquisition, I will plunge at once into the thick of them by the introductory premise that "Watt's Crystal Gold" is not to be confounded with any of the other so-called crystal golds which we have offered us from time to time. It is, as superbly shown under the microscope, an interlaced mass of perfect golden fern leaves, which have been grown up from, and in the continually saturated solution of gold, by a process of electrical action. While all others that I have ever examined show no organization whatever, but are a mere agglomerated mass of gold particles, which cohere temporarily like wet sand, and are, moreover, produced by chemical action, mercury, etc., methods which were experimented with and abandoned by Watts at least 25 years ago.

I have used this beautiful form of gold exclusively for something more than fifteen years, and associated with other golds, before that time, and so say what I do know, that all operations which can be accomplished with any other gold, or by any other operator, can be at least as perfectly accomplished with this, and with less expenditure of force and strain, and indeed in every sense, with far greater ease of manipulation than with foil golds. Crystal gold possesses moreover some excellencies which do not obtain in foils, one of which is the steel-like hardness of surface of the fillings produced with it, which in grinding surfaces is so especially valuable for their resistance to, and withstanding of, the battering force of mastication, and of the cusps of opposing teeth, and in the exquisite perfection with which it adapts itself under honest manipulation to the wall of the cavity, indeed so close and perfect is it, that delicate pointings of the microscopic crystals have been shown to have entered open ends of the dentinal tubuli themselves. This waxy adaptiveness would be, under any circumstances, a most valuable attribute, but it becomes doubly so when taken in connection with the fact of the gold being condensed with so little force as compared with foil. In such circumstances, for instance, as very frail front teeth, where for appearance sake, it is often expedient to save every atom of even thin remaining enamel, the value of this quality in crystal gold is inestimable, and I wish to be perfectly understood in regard to condensation, in such circumstances of frail walls as just *instanced*. The filling should be of course, and is, with faithful manipulation of this gold with small points, practically as closely adapted and as well consolidated against these walls and throughout the stopping, as if against strong walls, and all this where a proper condensation of foil would be far more likely than not, to rupture the enamel, even granting to the foil the skillful manipulation. If crystal gold can be depended upon to do this so delicate and refined work among "egg shells," what must be its capacity for easy and perfect adaption in the great mass of the usual cavities having strong walls. I deem it a work of supererogation to enlarge upon, or even do more than suggest the point to intelligent professional gentlemen. This wonderful combination of all the compliant and waxy characteristics of "soft" foil with even more of the cohesive quality than is possessed by most cohesive golds, and superadded to this, a very hard finished surface peculiarly its own, are the especial characteristics of Watt's Crystal Gold, and from such a superb *tout ensemble* for gold filling material as that, the application of the motto "multum in parvo" seems especially appropriate.

As I have previously said, I have used this form of gold exclusively for more than fifteen years, and although I always have a quantity of other gold in my gold drawer, and, get most new varieties as they appear, and keep them more or less even on my trays, because I do protest that I despise the prejudiced and obstinate riding of a hobby, and always feel ready to use any gold or any material that I can produce the best results with, in any and each individual case. I am nevertheless wholly unable, as a general rule of every day practice, to find any place in any activity where I can possibly employ them to any advantage as compared with crystal gold, and am constrained to store away the pellets and cylinders, etc. in the body of large stoppings from time to time, *to get rid of them.* "Aha" I hear some captious critic say "so you *do* manage practically to combine foil with your crystal goid after all." Don't think so gentlemen, I merely and truly, as I say, having kept some foil preparations for a time, I seek to dispose of the—to me—useless material, by putting it where, possibly many of you would say, it would "do the least harm," in the centre of a large stopping, and precisely as I would stow away pieces of crystal gold which had become in any way somewhat condensed, and. in my opinion, unfit, to place next the walls. One exception, however, to my habit, as a mere matter of extra prudence, of binding together a largely built out restoration in gold, with occasional strips of heavy foil, feeling that the magnificient hardness of condensed crystal gold might possibly militate against the toughness and consequent integrity of a long, thin restoration. Beside this negative proposition as to my employing no other gold, I wish to place the positive one, that I *do*, with *very* rare exceptions use, only this; that is to say, so far from employing it only for usual operations, and then plastering up all the difficult or large ones with amalgam, I almost never—probably not nearly as often as I ought—employ amalgam, or other plastic filling materials for permanent teeth, and as seldom order teeth out. And I say all this in order to make the point, and to make it plain, that I *never hesitate about doing any operation whatever with crystal gold*, that I depend upon it for all and any dental stoppings or restorations whatever, small and great, easy and difficult, and that if any failure occurs (and I am not, I hasten to assure you, one of those transcendent operators who never, never have them,) I *know* the failure to be consequent upon my, somehow or somewhere defective manipulation, and not any fault of the gold employed. For instance, if I were to "take a piece as large as the cavity" (as, for many years the instructions accompanying it directed, and which some operators who use it, apparently do now,) and jam and plaster it into the cavity trusting to some magic of affection in the gold to seek out the walls of the cavity and cling to them, I should fully expect, as a consequence, the leaky and worthless stopping that is so often seen, and which advertises to all who see it, not the incompetency of the gold, but of the operator who essayed to place it.

Notwithstanding the beautiful, even silky, softness and pliancy of crystal gold as it takes it place in the cavity, it becomes hard under manipulation, and that very quickly, and being cohesive to the last degree, must be treated after this first placing in the cavity like cohesive foil, and, as I have said in a previous paper, if crystal gold be given the same honest care that a conscientious operator would in like circumstances apply to cohesive foil, he would surely produce the same perfect stopping, and in addition receive the benefit of the grand combination of the special beauties and advantages in both soft and cohesive golds.

It is absolutely imperative that the first pieces of crystal gold be held immovably in the position in which they have at first been placed, and retaining points, or an angle of the cavity may be employed for the purpose. My own very decided preference, however, and the method which I employ wherever possible, is to arrange to hold the completed stopping by the general shape of the cavity, rather than by means of the deep pits so often employed, as I greatly deprecate any so unnecessary reaching out toward the pulp with conductive material, as are these retaining points when filled. In this case the first pieces may be held accurately to their position, by means of a pointed instrument in the left hand, continuing its employment till in condensing these first pieces, and proceeding with the stopping, the shape of the cavity is found to hold the gold securely without it. Condensing each piece finally as one progresses, exactly as a careful operator would proceed with cohesive foil, for as I have before remarked crystal gold, *when first applied to the cavity*, is waxy and adaptive as is the softest of soft foils; but there its similarity ends, as it should, and this first application being once made, it must be treated thereafter precisely as though it were cohesive foil.

A good operator when applying a cohesive foil pellet to the cavity would not proceed to disintegrate it by punching it full of holes with a sharp point; neither would he on the other hand attempt to model large thick pieces of it into position with "spoon" modellers and expect anything but a worthless uncondensed cavityful of something akin to loose shavings or sheet iron scraps as a result. Neither would he jam a large mass of cohesive foil into a cavity, and by thrusting wedge shaped instruments down through it, expect to force it to spread out sidewise and form the perfect adaptation to the walls of the cavity which the merest professional child ought to know is the *sine qua non* to the saving stopping, and yet gentlemen do all of these things, commit all of these sins against crystal gold, and cry all maledictions upon this gentle material, for the results, forsooth. "You miserable lamb," says the wolf, standing in the stream above him; "how you have roiled up and spoiled this water for me. I will therefore destroy you."

If gentlemen would but--for instance—take the cake of gold carefully and gently between the fingers of the left hand, and, with long pointed tweezers in the right, as carefully push off-without too close approximation of points of the tweezers—a tray full of small pieces, and then, the rubber dam being in position on the tooth, of course, the cavity dry, etc.—take up one of the pieces on the end of such filling instrument as would be employed were cohesive foil pellets being used (in any shape, so that 'tis small, and is very finitely serrated,) carry it through the annealing flame, and to the position in the cavity where it is deisgned to commence the stopping, hold it absolutely still, until it has been fully and entirely condensed, employing any means of condensation which would properly consolidate pellets of cohesive foil, add other pieces, in the same manner, still holding the compacted portion immovable, until it will of a certainty hold itself, adding no single piece however till that already in the cavity has been fully and finally consolidated, never changing the position of any pellet after its first application to its place, using so small pieces as to be *sure* that no piece is added which, from its size or thickness, they could not, with absolute certainty, condense throughout its substance, without chopping or punching it full of holes, packing directly against the walls of the cavity, and remembering that no cohesive gold spreads or wedges out under manipulation like soft foil, and that consequently all packing must be done directly upon the part desired to be compacted.

In a word, proceeding throughout the operation precisely as though it were cohesive foil pellets which were being employed, and, my word for it, they will be surprised and delighted to find how they would be accomplishing the same results with this gold as with foil, and with how much greater ease and pleasure and delicacy they where doing it.

If then they continued to the end of the placing of the gold and polishing and finely finished the exposed surface, they would find in that such resisting steel-like hardness as is implied in the fact that a patient will return days afterwards to have a mere nothing taken off from the surface; *they can't hammer it down*, slightly excess tho' it be,

My own preference has always been for No. 1, in contradistinction to the more condensed Nos. 2, 3 and 4, and for such delicate handling of even this, as that it shall certainly *be* No. 1, when it reaches the cavity, and not have been handled and pressed into the equivalent of No. 3 or 4; as the very essence of the value of crystal gold—to my mind—lies in the tremendous difference in bulk between the exquisitely soft silky state in which it comes to us, and its finally consolidated one in the cavity, and that the great change taking place under the guidance of the instrument of the operator implies all of the possibilities of the marvelously close adaptation to the walls of the cavity of which I have spoken, but which superb attribute of crystal gold is deliberately thrown away, to start with, by using the condensed Nos. 2, 3 end 4, and equally so, by the careless handling and squeezing of No. 1. In short I want to do all of the condensing *in the cavity*.

The ultimate destination of all, with our varied methods and golds, is the goal of the perfect stopping or restoration, and each being devoted to his own method, and becoming skiilful in it, it becomes *his* best one, and we may earn much from these specialists, even tho' we ourselves may either not be specialists, or that we even do not at all employ his particular method.

The soft-foil operator teaches us much about adaptation. The heavy foil man—I'm a little lame here—perhaps the want of it. The cylinder operator about rapidity of operating. The electric foil man lauds its lead-like ductility. This cohesive foil man his general power to restore beyond the cavity, *chacun a son gout.* But it is obviously unfair for one man to test another's method or material by his own manipulations, and then depreciate Pegasus, because forsooth he don't yoke with his ox!—*Arch. of Den.*

